# The impact of immune checkpoint inhibitors in patients with chronic lymphocytic leukemia (CLL): A protocol for a systematic review and meta-analysis of randomized controlled trials

**DOI:** 10.1097/MD.0000000000021167

**Published:** 2020-07-10

**Authors:** Aviwe Ntsethe, Phiwayinkosi Vusi Dludla, Tawanda Maurice Nyambuya, Siphamandla Raphael Ngcobo, Bongani Brian Nkambule

**Affiliations:** a School of Laboratory Medicine and Medical Sciences (SLMMS), College of Health Sciences, University of KwaZulu-Natal, Durban, South Africa; b Department of Health Sciences, Faculty of Health and Applied Sciences, Namibia University of Science and Technology, Windhoek, Namibia; c Department of Life and Environmental Sciences, Polytechnic University of Marche, Ancona, Italy; d Biomedical Research and Innovation Platform, South African Medical Research Council, Tygerberg, South Africa.

**Keywords:** adverse events, chronic lymphocytic leukemia, immune checkpoint inhibitors

## Abstract

**Introduction:**

The global burden of chronic lymphocytic leukemia (CLL) has constantly increased over the years, with a current incidence of 3.5 cases per 100,000 people. Although the conventional drugs used to treat CLL patients have been effective treatment failure rate in some of the patients is alarming. Therefore, as a result, novel treatment strategies with improved outcomes such as the blockade of immune checkpoints have emerged. However, consensus on the risk-benefit effects of the using these drugs in patients with CLL is controversial and has not been comprehensively evaluated. This systemic review and meta-analysis provide a comprehensive synthesis of available data assessing adverse events associated with the use of immune checkpoint inhibitors in patients with CLL as well as their influence on the overall survival rate.

**Methods:**

This protocol for a systematic review and meta-analysis has been prepared in accordance with Preferred Reporting Items for Systematic Review and Meta-Analysis Protocols 2015 guidelines. A search strategy will be developed using medical subject headings words in PubMed search engine with MEDLINE database. The search terms will also be adapted for gray literature, Embase, and Cochrane Central Register of Controlled Trials electronic databases. Two reviewers (AN and SRN) will independently screen studies, with a third reviewer consulted in cases of disagreements using a defined inclusion and exclusion criteria. Data items will be extracted using a predefined data extraction sheet. Moreover, the risk of bias and quality of the included studies will be appraised using the Downs and Black checklist and the quality and strengths of evidence across selected studies will be assessed using the Grading of Recommendations Assessment Development and Evaluation approach. The Cochran's Q statistic and the I^2^ statistics will be used to analyze statistical heterogeneity across studies. If the included studies show substantial level of statistical heterogeneity (I^2^ > 50%), a random-effects meta-analysis will be performed using R statistical software.

**Ethics and dissemination:**

The review and meta-analysis will not require ethical approval and the findings will be published in peer-reviewed journals and presented at local and international conferences. This review may help provide clarity on the risk-benefit effects of using immune checkpoint inhibitors in patients with CLL.

**Systematic review registration:**

International prospective Register of Systematic Reviews (PROSERO) number: CRD42020156926.

## 1 Introduction

The global incidence of leukemia has significantly increased over the years, with chronic lymphocytic leukemia (CLL) cases having a higher prevalence compared to all other lymphoid malignancies.^[1]^ Although the exact aetiology remains elusive, age, lifestyle and environmental factors have been identified as some of the major consequences implicated in the development of CLL.^[2,3]^ To date, it is well established that CLL is the most common type of leukemia, accounting for approximately 37% of all cases of blood malignancies,^[4]^ with an average global prevalence of about 3.5 cases per 100,000 people.^[5]^ In Africa, statistics on the incidence of CLL is very limited with isolated studies reporting on this form of leukemia.^[6–11]^ Nonetheless, various therapeutic drugs including those that modulate the function of immune checkpoints receptors are continuously being developed and their effectiveness tested in the management of patients with CLL worldwide.^[12–14]^

Immune checkpoints regulate immune function and play a crucial role in preventing autoimmunity.^[15–17]^ However, in CLL, the signaling of immune checkpoint receptors is dysregulated which results in immune dysfunction.^[18,19]^ Briefly, CLL is a monoclonal disorder that is characterized by the accumulation of functionally incompetent B-cells with a distinctive CD19^+^, CD20^+^, CD5^+^, CD23^+^ lymphocyte surface markers and surface immunoglobulin-positive phenotype in the peripheral blood, bone marrow, and lymph nodes.^[20,21]^ Hence, anti-CD20 monoclonal-based drugs such as rituximab and ofatumumab are used as standard treatment for CLL.^[12,22,23]^ However, these drugs are associated with severe adverse events such as neutropenia and thrombocytopenia,^[24–26]^ with others reporting on their ineffectiveness as monotherapy.^[27]^ Thus, the need to urgently broaden our understanding of the pathophysiological mechanisms implicated in the aggravation of CLL.

Although CLL is a B-cell malignancy, recent studies have also described the involvement of T-cells in the pathogenesis and progression of the disease.^[28–30]^ In fact in CLL, T-cell exhaustion mediated by an upregulation of coinhibitory receptors such as programmed death-1 (PD-1), lymphocyte-activation gene 3 (LAG-3), T-cell immunoglobulin-3 (TIM-3), and cytotoxic T-lymphocyte-associated protein 4 (CTLA-4) has been reported.^[18,31]^ Consequently, this has led to the advancement of immune checkpoint inhibitors that targets both B and T-cell function as a treatment strategy for CLL.^[32]^ However, contradictory findings on the effects of using immune checkpoint inhibitors in CLL patients have been reported.^[13,32–36]^ Thus, the exact effect of immune checkpoint inhibitors in CLL is contradictory and needs to be investigated further. As a result, due to high quality of evidence reported in randomized controlled trials (RCTs), this review will target such studies to assess and update available literature on the impact immune checkpoint inhibitors in CLL.

## 2 Research question

What are common adverse events associated with the use of immune checkpoint inhibitors in patients with CLL?

## 3 Objectives

1. To assess the adverse events associated with the use of immune checkpoint inhibitors in patients with CLL.
2. To estimate the overall survival rate of patient with CLL on immune checkpoints inhibitors.

## 4 Methods

This protocol was prepared in accordance with the Preferred Reporting Items for Systematic Review and Meta-analysis Protocols (PRISMA-P) 2015 guidelines.^[37]^ In addition, the protocol has been submitted on PROSPERO for registration.

## 5 Eligibility

### 5.1 Study design

This systematic review and meta-analysis will include RCTs with a clearly defined population and interventions used. While, observational studies, reviews, case studies, and animal studies will be excluded in this study.

### 5.2 Participants

Studies evaluating the use of immune checkpoint inhibitors as a treatment method in adult patients (≥18 years) with CLL, will be included.

### 5.3 Intervention

We will include studies reporting on the use of immune checkpoint inhibitors targeting PD-1, CTLA-4, LAG-3, and TIM-3 signaling as a therapeutic strategy for CLL.

### 5.4 Comparators

CLL patients on immune checkpoint inhibitor drugs that did not develop any associated adverse events.

### 5.5 Outcomes

The primary endpoints will include the following:

1. Adverse events that are associated with the use of immune checkpoint inhibitors. These include mortality, endocrinopathies, and dermatitis, autoimmune, gastrointestinal and hematological disorders.

### 5.6 Surrogate outcomes

1. Overall response, progression-free survival, and event-free remission
2. Common low-grade adverse events as described by National Cancer Institute grading system^[38]^

### 5.7 Search strategy

The search strategy will be developed using medical subheading words on MEDLINE and will be adapted to gray literature, Embase, The Cochrane Central Register of Controlled Trials databases, and ClinicalTrials.gov with the help of an experienced librarian. The search strategy will consist of the following keywords and their respective synonyms; chronic lymphocytic leukemia, anti-PD-1 drugs (nivolumab, Pembrolizumab, Pidilizumab, Atezolizumab, Avelumab), anti-PD-L1 drugs (Atezolizumab, Avelumab, Durvalumab), anti-CTLA-4 drugs (Ipilimumab, Tremelimumab) anti-LAG-3 and anti-TIM-3 drugs and adverse events.

### 5.8 Study selection

The study screening and selection process will be carried out by 2 independent reviewers (AN and SRN) to eliminate risk of bias and inconsistencies with regards to reviewers’ inclusion and exclusion of studies. Each reviewer will screen tittle, abstract, and full texts in contrast to the inclusion criteria. The exclusion criteria for title and abstract screening phase will include duplicate of the same study, reviews, observation studies, and studies that reported nonimmune checkpoint-related CLL therapeutic drugs. In cases of disagreements, a third reviewer (TMN) will be consulted for arbitration. The level of inter-rater agreement will be determined by using the Cohen's kappa inter-rater reliability.^[39]^ A kappa value of < 0.00 will be interpreted as a poor strength of agreement, 0.00–0.20 as slight agreement, 0.21–0.40 as fair agreement, 0.41–0.60 as moderate agreement, 0.61–0.80 as substantial agreement, and 0.81–1.00 as perfect agreement.

## 6 Data management

### 6.1 Data collection process

The reviewers (AN and SRN) will develop a data extraction form that will be used in the collection relevant data items. To reduce data entry errors, selected studies will be independently assessed by two reviewers (AN and TMN), the third reviewer (BBN) will be consulted for arbitration in case of any disagreements.

### 6.2 Data items

Extracted data items will include the author's name, year of publication, sample size, duration of follow-up, outcome measures, age, gender, immune checkpoint receptors targeted by the drugs, dosage, adverse events reported, and overall survival rate.

### 6.3 Data simplification

Studies will be grouped according to the type of immune checkpoint inhibitor used. In addition, studies will be grouped based on the immune checkpoint receptor targeted (PD-1, PD-L1, CTLA-4, LAG-3, TIM-3). Studies that report on immune checkpoint inhibitor combined with other conventional drugs will be pooled. The adverse events will be grouped and graded into grades 1 to 4 based on their severity. Group considered 1 and 2 are mild and moderate whilst groups 3, 4, and 5 are severe.^[38]^

### 6.4 Risk of bias in individual studies

To evaluate the potential risk of bias in RCTs, Cochrane collaboration tool for assessing bias^[40]^ and Downs and Black checklist^[41]^ will be used. Two independent reviewers (AN and SRN) will appraise all included studies and a third reviewer (PVD) will be consulted in cases of disagreements.

### 6.5 Data synthesis

A summary of findings table will be used to provide a synthesis of the main outcomes of included studies. Moreover, if the included studies are homogeneous in terms of the type of immune checkpoint inhibitor used and participant characteristics, data will be analyzed with Rev Manager (Version 5.3) to conduct a meta-analysis. To measure statistical heterogeneity between studies, I^2^ and Chi squared statistical tests will be used.^[42,43]^ An I^2^ value of > 50% will be considered substantial heterogeneity.^[44]^ To find the sources of heterogeneity within the included studies, a subgroup analysis and meta-regression comparing the study estimates from different study-level characteristics, quality, intervention type (type of immune checkpoint inhibitor), and the reported effect measure of adverse events will be conducted.

## 7 Quality assessment of the cumulative evidence

The Grading of Recommendations, Assessment, Development and Evaluation assessment tool^[45]^ will be used to assess the overall quality of evidence. Moreover, the quality of each included study will be independently evaluated by 2 authors (AN, SRN). The third author (TMN) will adjudicate in cases of disagreements. The quality of evidence will be assessed based on several factors such as study limitations, indirectness of results, and publication or reporting bias. The evidence of each outcome will be rated as high, moderate, low, or very low.

## 8 Discussion

Immune checkpoint inhibitors have been shown to be effective in the treatment of CLL, its association with adverse events is controversial^[13,32]^ and has not been critically assessed. Therefore, this systemic review and meta-analysis aims to evaluate the risk-benefit of using immune checkpoint inhibitors as a therapeutic strategy for patients with CLL. Findings from this study will give a better understanding on the effectiveness of immune checkpoint inhibitors as well as paving way for strategic development of effective therapies and management of patients with CLL.

## Author contributions

AN, TMN, PVD, and BBN conceptualized, designed, and drafted this manuscript. All authors including SRN wrote and approved the final manuscript. BBN is the guarantor of the review.
